# Gamification as a Promoting Tool of Motivation for Creating Sustainable Higher Education Institutions

**DOI:** 10.3390/ijerph19052599

**Published:** 2022-02-23

**Authors:** Johanna Andrea Navarro-Espinosa, Manuel Vaquero-Abellán, Alberto-Jesús Perea-Moreno, Gerardo Pedrós-Pérez, Maria del Pilar Martínez-Jiménez, Pilar Aparicio-Martínez

**Affiliations:** 1 Unidad de Seguimiento a Graduados, Banca Laboral y Prácticas Preprofesionales, Universidad de ECOTEC, Guayaquil 090501, Ecuador; jnavarro@ecotec.edu.ec; 2 GC12 Clinical and Epidemiological Research in PrimaryCare, Instituto Maimónides, Campus de Menéndez Pidal, Universidad de Córdoba, 14071 Córdoba, Spain; n32apmap@uco.es; 3 Departamento de Enfermería, Fisioterapia y Farmacología, Campus de Menéndez Pidal, Universidad de Córdoba, 14071 Córdoba, Spain; 4 Departamento de Física Aplicada, Radiología y Medicina Física, Edificio Albert Einstein, Campus de Rabanales, Universidad de Córdoba, 14071 Córdoba, Spain; aperea@uco.es (A.-J.P.-M.); fa1pepeg@uco.es (G.P.-P.); 5 Responsable Grupo Investigación PAIDI de la Junta de Andalucía TEP149, Modelos de Simulación en Energías, Transporte, Física, Ingeniería y Riesgos Laborales, Edificio Albert Einstein, Campus de Rabanales, Universidad de Córdoba, 14071 Córdoba, Spain

**Keywords:** health and sustainable environments, gamification, higher education institutions, motivation

## Abstract

Higher Educational Institutions (HEIs) are responsible for creating healthy and sustainable environments for students and teachers through diverse educational paradigms such as gamification. In this sense, the Healthy People 2030 and the Sustainable Development Goals indicated the imperative to provide inclusive and equitable quality education to promote a healthy environment and life. The principal objective was to analyse the impact of gamification on health development in HEIs, highlighting their positive and negative effects. To achieve such an objective, a bibliometric analysis was carried out. The 257 documents showed no significant increasing trend in the last decade (*p* > 0.05) related to the pandemic. Most of the publications were conferences (45%), and the few published articles were the documents with more citations (*p* < 0.001). According to their index in Journal Citation Reports, there were significant differences between the citations of articles published in journals (*p* < 0.001). The analysis of journal co-citations showed that the leading journals (such as *Computers in Human Behavior*) had a significant part in the clusters formed (*p* < 0.001), conditioning also the keywords, especially the term “motivation”. These findings were discussed, concluding that the experimental studies focused on the teachers’ adverse effects are yet to come.

## 1. Introduction

Higher Educational Institutions (HEIs) are responsible for creating healthy environments for students and teachers [1]. This function, framed by the Health Promoting University definition, focused on encouraging health in HEIs, considering the relevance of sustainability, from the quality of education to adequate mobility [2]. This approach creates sustainable organisational modifications that generate circumstances supportive of health and well-being in line with an ecological view of health [3]. This understanding of sustainability connected to the concept of a Health Promoting University is characterised by education and research features supporting innovation reinforced by proper evaluation [4]. Such an approach has been emphasised since the late 1990s in different healthcare colleges and centres across countries [3], highlighting students’ academic and personal development and a healthy, supportive workplace for teachers [5].

Additionally, HEIs are dynamic agents of change for sustainable development; teaching informal contexts contributes to the training of professionals in the 21st century [6]. The use of project-based pedagogies for sustainability allows them to develop critical, reflective, creative, and resilient thinking skills as well as attain greater mastery of ICT to engage and respond socially to the needs of their interconnected environment [7]. For students, academic success and personal growth are critical factors in the self-esteem and self-perception of students [8,9], confirming their worthiness and emotional and mental health for the rest of their lives [10,11]. Academic achievement is co-dependent on health problems that positively or negatively impact younger people’s or children’s well-being [12]. One example of this association is physical activity, whose higher frequency increases academic performance [12].

Meanwhile, this academic performance is also linked to the educational quality, which needs to adapt to students’ needs and requirements of the HEIs, being framed in the Sustainable Development Goals [13,14]. Such quality depends on the technological tools and adequacy of the workplace for the teachers’ skills, training, and knowledge [15,16]. Additionally, these tools, also known as information and communication technologies (ICTs), can cause teachers stress, technostress, and anxiety, among other emotional or mental distress problems [6,17]. Despite these side effects, HEIs have integrated the utilisation of ICTs since they are used on a daily basis by students and result in greater connection or feedback between students and teachers, incorporating better knowledge and improving the students’ academic performance [7,18]. The negative or positive impact of these tools on the health of the different agents (students and teachers) in HEIs and their healthy environments depends on diverse factors from the type of educational paradigms, such as gamification or serious games, the implementation of sustainable education, the safety and occupational health measures for the teachers, the integration of the new strategies for creating healthy environments, or change in the design of education from face-to-face to online teaching [6,19,20,21].

## 2. Social and Sustainable Development, Gamification, and Health

Numerous studies, as well as organisations [22,23,24], have indicated the need to provide a high quality of education, being even framed in the Sustainable Development Goals (SDGs) [22] and defined as the 4th goal in the Sustainable Agenda 2030 [23]. In this sense, different goals from the Healthy People 2030 [24] and the SDGs [25] indicate an imperative to provide inclusive and equitable quality education to promote a healthy environment and life. Moreover, the U.S. Department of Health and Human Services indicated that education could prevent diverse illnesses, such as obesity or hepatitis, the pinnacle of the prevention of the 4th grade [26]. Based on all the data, education is critical for cultivating sustainable, healthier social environments [23,24,27,28]. The role of education in creating such environments seems to be based on its capacity to transform people’s lives by integrating values, knowledge, skills, and a global vision towards social welfare [27]. Therefore, it is imperative to include critical thinking, systematic reflection, collaborative working, and students’ responsibility from a practical, multidisciplinary, and comprehensive approach [29].

Several authors have indicated how integrating ICTs in education promotes healthy development [30,31], including the self-perception [9,32]. In this context, new active methodologies and pedagogical strategies focused on ICTs, mainly serious games in science, technology, engineering, and mathematics (STEM), improve the motivation and self-esteem of students [33,34,35]. In HEIs, the use and implementation of games, also known as gamification, has aimed to motivate and create interest in students [36,37] through training experiences [20]. The teaching approach has increased in the last decade based on the ease and little programming required, and previous training and knowledge [20,38,39], despite being theorised and hypothesised since the 1990s. One reason for the popularity of gamification in higher education seems to be the improvement of motivation, flow, skills, and learning perceived by both the teachers and students [36].

Another reason could be explained by online teaching, known as e-learning, or a combination of face-to-face with online education, known as b-learning, based on using ICTs and games as basis instruments for the teaching process [40,41].

Incorporating ICTs in educational institutions to strengthen different modalities, from e-leaning to face-to-face, has taken place little by little as a continuous improvement process [42]. However, with the appearance of COVID-19, educational institutions were forced to generate pedagogical and technological strategies that would allow them to give continuity to current programs. Educational institutions needed to abruptly migrate to e-learning, including the ICTs, without understanding the positive (such as motivation) or negative (such as level of stress and anxiety) effect [43].

As a result of the COVID-19 pandemic and how most studies focused on elementary and middle schools [42], the role and impact of gamification in HEIs during the last two decades seems to be undervalued. Therefore, the objective of this research was to analyse the current scientific knowledge regarding the impact of ICTs, specifically gamification, on health development in HEIs in the last two decades, highlighting the positive and negative effects. In addition, a secondary objective was to determine the impact of ICTs and gamification on teachers as active agents in HEIs and their current growth in STEM fields, especially in medical areas.

## 3. Materials and Methods

### 3.1. Design of the Study

The inclusion, use, and implementation of ICTs have positively impacted the teaching–learning process and allowed the incorporation of new methodologies such as gamification and game-based learning, increasing the motivation and interest of students using tools, applications, and virtual laboratories, the latter widely used in STEAM disciplines [20].

Based on the previous description, bibliometric studies have become an effective tool for determining the quality of current scientific knowledge and its impact on the field of education [44,45,46]. Bibliometric studies contextualise scientific information at a national and international level, contributing to understanding the relationship between information and communication technologies and the education field [47,48]. As pointed out by Harman et al. [44], citations and analysis of previous works are an essential contribution in any field, especially in education. Harman et al. [44] and Trinidad et al. [45] analysed the impact of gamification in the education field from different time periods (from 2010 to 2013 and 2011 to 2019). Both time frames focused on the inclusion and initial use of gamification in education [49], although the ICTs in education have been included since the early 2000s [43]. Moreover, these studies [44,45] focused only on gamification, and other possible ICTs were excluded in the educational field without distinctions. The current research was designed to provide further insight into two aspects: the cover of an underrated topic, based on ICTs, especially the gamification, in STEM education, including the health field, and a combination of qualitative and quantitative analysis. To achieve both insights, a deeper research based on a bibliometric design focused on ICTS, emphasising gamification in the STEM field, including health education, based on the last two decades and with specific research questions, was carried out. The research questions (RQs) and workflow were set according to Zupic and Cater [50]:

RQ1: Which is the publication trend for ICTs, especially gamification, in STEM education?

RQ2: Which countries and journals contribute to this field, and what is their relationship?

RQ3: Which are the top publications and their impute on ICTs and gamification in STEM education?

RQ4: How has the knowledge in this field grown over the last two decades?

RQ5: How have the research focus and major topics evolved in the timeframe?

RQ6: What influence do ICTs and gamification have in health education as a subsection of STEM education?

The procedure of this research followed the recommendations of previous researchers [51] following the five-step research design (with the design questions and selection of bibliometric and visualisation methods), compilation of the bibliometric data (select the databases and execute the searches with the filters), analysis (bibliometric methods and use of supportive tools), visualisation, and interpretation.

### 3.2. Selection of the Databases and Design of the Research: Exclusion and Inclusion Criteria

The information search was carried out in the Web of Science (WOS) and Scopus databases to obtain further information and reduce the possible bias of selecting documents [52]. The bibliographic search was carried out without using the terms Medical Subject Heading (MeSH), since the keywords identified according to the objectives of the present investigation were not determined according to their definition (Table 1). The reason was based on game theory which is a discipline that impacted the 1950s and 1960s, was created by Nash in 1925, and achieved consolidation in different areas of knowledge (mainly administrative ones) and resolved real-world problems [53]. Additionally, the term “gamification” has been modified in the last decade, and newly derived methodologies applied to education in 2010 [49] and game-based learning in 2017 and linked to ICTs have generated a change in the educational paradigm, which are terms that due to their current relevance were considered in the bibliographic search. Additionally, “ICT” terms were used to identify the tool, and “gamification” was implemented for the educational paradigm, resulting in data based on games as educational tools for healthy environments. The Boolean operators used were “OR” and “AND” to link the terms and identify the “title”, “abstract”, and “research” framed in the research questions.

The exclusion criteria used included the following. First, the period to produce the documents being selected covered only the last twenty years, since the ICTs have been included in education in the last two decades [42], and gamification has changed in the last decade as well [49] being framed in the Decade of Education for Sustainable Development (DESD) [54]. In addition, articles focused on the elementary and middle educational institution; focused on students’ perspectives or health problems not related to the educational paradigm or ICTs in their education; studies based on general education with the perspective of patients or other end-users; and finally studies that lacked analysis of ICTs’ role were excluded as well. Additionally, the document type was determined to exclude non-scientific productions, such as projects. The data were analysed and selected or eliminated according to the year of publication, journal, keywords, title, and abstract based on the exclusion criteria.

### 3.3. Research Strategies: Exploratory and Final Research

In December 2020, the exploration of related terms began where different combinations included new technologies, ICT or TIC gamification, serious games focused on Higher Education; however, being global terms, the results obtained did not fit the required approach. The primary search strategy in each database was implemented according to the keywords selected in January 2021. The initial search was carried out only in WOS with the following terms TI = (“TIC”) OR TI = (“ICT”) OR AB = (TIC) OR TI = (ICT) OR AK = (TIC) OR TI = (ICT) AND TI = (GAMIFICATION) OR TI = (LUDIFICATION) OR TI = (SERIOUS GAME) OR TI = (GAME-BASED LEARNING) OR AB = (GAMIFICATION) OR AB = (LUDIFICATION) OR AB = (SERIOUS GAME) OR AB = (GAME-BASED LEARNING) OR AK = (GAMIFICATION) OR AK = (LUDIFICATION) OR AK = (SERIOUS GAME) OR AK = (GAME-BASED LEARNING) AND TI = (HIGHER EDUCATION) OR TI = (UNIVERSITY) OR AB = (HIGHER EDUCATION) OR AB = (UNIVERSITY) OR AK = (HIGHER EDUCATION) OR AK = (UNIVERSITY). As a result, 94,908 documents were obtained and downloaded in various csv document formats for later analysis.

**Figure 1:**
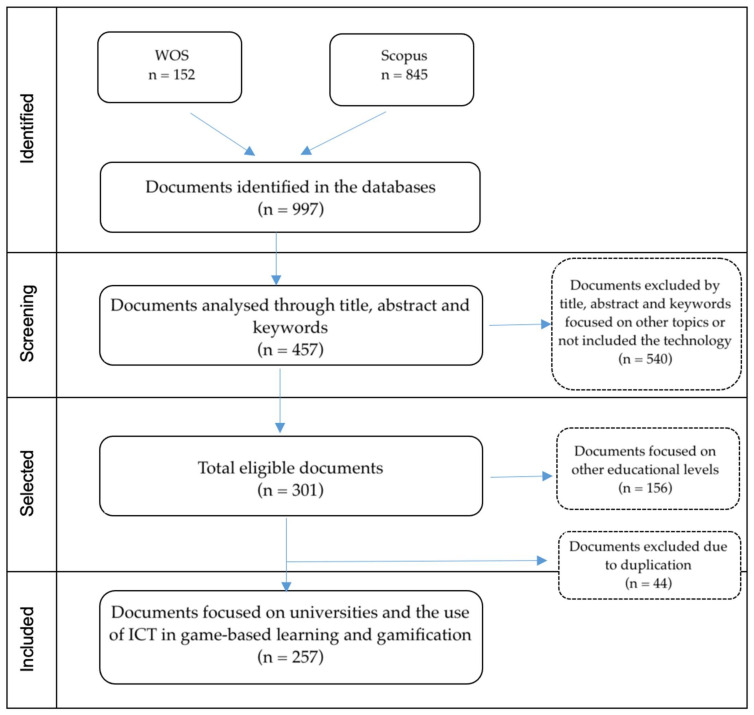
Flow diagram of the selection of articles for the quantitative analysis.

The results obtained in the exploratory research strategy were linked to the term ICT and its term in Spanish. The great number of results and its heterogeneity indicated that the initial search was too wide for an adequate analysis. It was observed that the term TIC is a term that has different connotations because it is used to abbreviate meanings related to different categories of the database [18]. An example of this issue was that the term TIC also included the Materials Science Multidisciplinary, Metallurgy Metallurgical Engineering and Chemistry Physical reference TiC powders (titanium carbide powder). Their different combinations, Engineering Electrical Electronic, is related to the investigation of materials fused with cobalt (Co-TiC), and materials for electrical conductivity (titanium carbide (TiC)) in Psychiatry and Clinical Neurology are commonly related to research on Tourette Syndrome and Tics disorders in children and adolescents. Based on the results, in June 2021, a new search was carried out, considering the exclusion of the ICT abbreviation used for Information and Communication Technologies in Spanish (ALL = ((ICT) AND (GAMIFICATION OR SERIOUS GAME OR GAME-BASED LEARNING) AND (HIGHER EDUCATION OR UNIVERSITY)). In this month, the research was implemented in the Scopus database (TITLE-ABS-KEY (ICT) AND TITLE-ABS-KEY (GAMIFICATION OR SERIOUS-GAME OR GAME-BASED-LEARNING) AND TITLE-ABS-KEY (HIGHER-EDUCATION OR UNIVERSITY)). As a result, 152 documents were obtained in Scopus and 845 documents were obtained in WOS, which were exported in Excel and bibliographic formats (csv and enw), including fields such as author(s), type of publication, title, abstract, keywords, year of publication, language, number of citations, unique identifier, and funding agencies. In the search, 997 documents were identified and subsequently analysed from their title, abstract, and keywords. During the analysis, 540 documents were eliminated, since they focused on aspects unrelated to the object of the current research, such as the perception of teachers or levels of satisfaction related to students. In addition, 156 documents were identified as linked to research focused on initial, primary, and secondary education levels, which were excluded. Other 44 duplicate documents were removed using Endnote. Finally, 257 documents were included related to the use of ICTs in HEIs, which is linked to studies that include gamification or game-based learning (Figure 1).

### 3.4. Analysis: Supportive Tools

The results from the research were analysed initially using descriptive analysis, such as the frequencies of documents per country and year, the language, primary sources, the field of the publication, the leading scientific institutions, associations among nations, the primary authors in the area, and the index keywords used. After obtaining all the data, SPSS program version 28 (IBM Corporation, Armonk, NY, USA), VOSViewer version 1.6.15 (Ness Jan van Eck, Leiden, the Netherlands), Excel version 17 (Microsoft Corporation, Redmond, WA, USA), and Endnote (Clarivate Analytics, London, UK) were used to analyse the information. The selection of these programs was based on the research questions being required, including descriptive analysis (Excel and SPSS), citation analysis (SPSS and VOSviewer), cross-reference (Endnote), bibliometric mapping (VOSViewer), and networking analysis (VOSViewer and SPSS). Meanwhile, the Bibliometrix program (Massimo Aria, University of Naples Federico II, Naples, Italy), which is an R statistical program for quantitative analysis in the scientometrics and bibliometric area, was not included, since the SPSS could provide further quantitative analysis [51,55,56].

The Excel program was used to identify and visualise the data, such as the frequency of publications per year or the number of documents each country produced. Endnote was used to eliminate the duplicated records and identify possible cross-references such as the studies from previous research fields [47,48], which was also included in the research of Trinidad et al. [45] and found in the final sample. The VOSviewer program used the csv format to identify and create mappings of co-citation, co-occurrence, and clustering between the authors, countries, and keywords, utilising a multidimensional analysis method. The SPSS program was selected for the citation analysis and the connection between countries, the evolution of the publications, and the relevance of quantitative indicators in this topic. 

The statistical analysis of the data was structured according to the quantitative or qualitative variables. The variables were structured according to the number of publications per country, institution and author, and citations. In addition, quantitative metrics (Journal Citation Report, quartile, and Journal Citation Indicator) were used to analyse the possible impact of published work and journal relevance. The descriptive analysis focused on relative frequencies, mean, and standard deviation (SD). For quantitative analysis of the 257 documents, the Mann–Whitney and Kruskal–Wallis tests and the Spearman’s correlation were used, based on the results obtained in the Kolmogorov–Smirnov test (*p* < 0.001). The Chi-square test for a sample indicated significant differences between countries to produce documents; additionally, the Cramer’s V test was used to determine the effect size for country and publications according to the years.

## 4. Results

### 4.1. General Results

#### 4.1.1. Publication Type, Language, and Trend (RQ1)

The results of the 257 documents showed that 35.9% of the available documents were articles, 45% were conference articles, and 18.7% were conference reviews. In recent years, there has been a growing trend of academic congresses where researchers present their scientific disclosures (with a maximum of pages). This response to the higher percentage of documents found that the mean of citations was lower (4.2; SD = 12.7) than those of other studies in different fields, which could be linked to the frequency of published papers. Moreover, the analysis of the citations per country (*p* = 0.12) and year of publication (*p* = 0.053) indicated no significant differences. This lack of significance could be associated with the dispersion of the data, being 93 documents from Spanish institutions and 164 from other institutions around the world.

These results matched a recent bibliometric analysis focused on gamification, which identified that 63% were conferences papers [45]. The central organisation and producer of the conference was the International Academy of Technology, Education and Development (IATED) (22.6% of the total documents) and the IEEE as the second organisation (6.2% of all the papers). Both results were fascinating, since IATED was created for any field focused on educational approach, indexed in Conference Proceedings Citation Index (Web of Science), and whose origin is based on the Polythetic University of Valencia [57]. Meanwhile, the Institute of Electrical and Electronics Engineers (IEEE) is a significant organisation with indexed journals, such as IEEE *Transaction on Education*, which strongly relates STEM education with interdisciplinary application [58]. Therefore, the conference papers seem to be associated with relevant editorial organisations. Regarding the language used in the research, English predominates in different international journals (92.4%), followed by publications in Spanish (6.8%), Russian, and Hungarian (0.4%), respectively.

**Figure 2:**
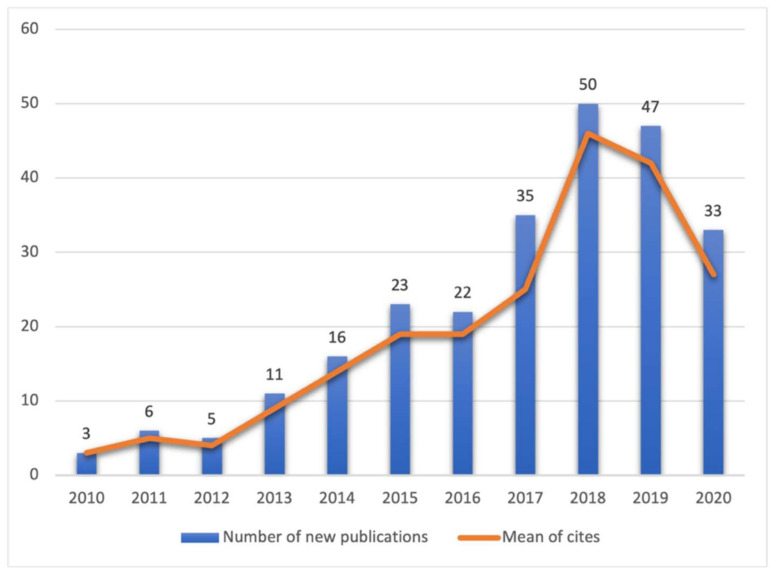
The number of documents per year and mean citations per year.

Figure 2 shows the frequency of academic publications related to ICTs, gamification, and STEM in higher education. There is a growing trend from 2013 to 2018, with 2018 and 2019 featuring more effective scientific communication from journals. Additionally, the median of the year of publication was set in 2018, which matched with the more substantial number of publications. In 2020, there was a decrease in the number of publications; this may be related to the still existing COVID-19 pandemic [59,60], which led to a change in focus from looking at gamification trends and their impact on STEM competencies due to experiences and good practices that will allow their contribution to the virtual modality.

These results showed how this specific research area continues to develop, since more publications are conference papers. The publication trend is experimenting with a light decrease, which could be explained by the decline in the publication in a specific area such as Engineering or the shift to experimental analysis. These results match those of Dégila et al. [61], which identified a decrease in e-learning in education publications and indicated how this decrease could be partially motivated by the COVID-19 pandemic. Nonetheless, the precious bibliometric analysis has not fully presented a possible reason for this research area’s decline.

#### 4.1.2. Publications and Collaborations between Countries (RQ2, RQ3, and RQ4)

The top five countries with more documents (Table 2) had significant differences (*p* < 0.01) regarding the number citations and documents, among which Spain was the leader (mean = 5.62; SD = 18.38; CI 95% = 9.39–1.83), followed by Italy (mean = 2.86; SD = 5.8), the United Kingdom (UK) (mean = 11.17; SD = 18.04), Croatia (mean = 0.5; SD = 0.71) and the United States (US) (mean = 3.2; SD = 7.47). The number of citations of the top five countries was 456 citations for Spain (46.0%), 40 citations for Italy (4.0%); 134 citations for the UK (13.5%), 5 citations for Croatia (0.5%), and 32 citations for the US (3.2%). In this sense, no significant differences were found between the citations per document and countries (*p* = 0.186).

Despite there being no significant difference for the citations, the country was linked to the Journal Impact Factor of the year of publication (*p* = 0.046) and 2022 (*p* = 0.012) as well as the percentile (*p* < 0.05). The number of citations per document was linked to being indexed in the Journal Citation Report (JCR) as an Emerging source or Social or Science Citation Index (*p* < 0.001). The data indicated that 12.8% were indexed, and 16.0% were included as Emerging sources, showcasing the difference between being indexed (*p* < 0.001) and not being indexed in the database (*p* = 0.04). These results manifest as previous research indicated that the JCR is a crucial point used by the authors to present their results and analysis.

Additionally, these findings are in sync with previous bibliometric articles in the educational field that indicated a high presence of European countries, especially Spain [45,61]. The initial relevance of European countries could be linked to the UNESCO initiatives from the Decade of Education for Sustainable Development and the fourth goal of sustainable development goals of the agenda 2030, which was related to leading [23,54].

Such results are also reflected by the top ten articles identified in this topic (Table 3), which are sorted by the number of citations. The ten articles with the highest citations were analysed according to the number of citations and the type of studies (Table 3). Based on the number of citations, among the ten most cited articles (Table 3), there are three observational studies, two theoretical studies or reviews, and five experimental studies based on a qualitative design. The results indicate that pre-and post-experiments based on qualitative design are the most common in this area.

Table 3 shows how the most relevant investigations (9/10) articles were published in Europe: five in Spain, two in the United Kingdom, one in Italy, and one in Germany; only one article was published in Turkey. In addition, it is reflected that the most cited articles are related to the applicability of game elements in the classroom. They were focused on understanding the essential elements to include in the design of the gamified components [62,63], academic performance [64], identifying their impact on learning process [65,66,67,68], motivation [69], and its effect on the development of skills [66,68,70], which are mainly linked to STEM education in the main text near the first time they are cited.

The table showed how eight publications were indexed in JCR and placed in the first or second quartile (Q1 and Q2). These results matched with the results from the Kruskal–Wallis test, being the tests statistic set at 51.86 and *p* < 0.001. The pairwise comparison and the adjusted *p*-values highly indicated a significant difference between not being indexed, being Q1 or Q2 (*p* < 0.001), and being Q1 and Q4 (*p* = 0.019). Moreover, the correlations indicated that a high quartile, such as Q1 or Q2, was associated with more citations (ρ = 0.427; *p* < 0.001). Another aspect that was linked to the citations and quartile was the Journal Citation Indicator (JCI) of 2020 that showed being over the mean of citations (*p* < 0.001). Additionally, the JCI was associated with the country; more differences between the significant country (Spain) compared to others were present (*p* < 0.001).

In addition, the data (Table 3) indicated that the most relevant articles were published in the 2010s; their thematic area were focused on Education, Multidisciplinary, or Health. These results were in sync with being indexed in the JCR and the year of publication, indicating that currently, in this area, there was no significant difference for the year of publication and being indexed (*p* = 0.053). Table 3 also indicated how most documents were published in Spain, focusing on the impact of gamification or other ICTs as motivational tools.

The top five articles regarding citations (Table 3) were carried out in Spain, Portugal, Turkey, and Scotland. These research studies were published from 2012 to 2018, matching the modification of the definition of gamification in 2010 and its posterior effect analysis in the HEIs [49]. Moreover, the top five articles were published in highly important journals, in the first and the second quartile, linked to the computing field.

The most cited article with 174 citations is from Spain, which shows that factors influence learning effectiveness through serious games [62]. This article focused on developing and evaluating a serious game set in the University of Alicante for English courses. The research was conducted using English Studies students, implementing a pre-and post-questionnaire with a quasi-experimental design. The article recognises the relevance of serious games in education. It highlights the importance of using immersive and interactive environments to help develop and improve skills, in this case, intercultural communicative competence, mainly achieving an adequate language level (middle-high level of English). This study indicated the effectiveness of gamification as an essential tool for future teachers or students. In addition, the article provides further factors, such as the observational impute of the end-users, that contribute to the inclusion of serious games in the learning process. The report states that games elements must include the learning results to be achieved and must be designed to promote student involvement.

The second article was a narrative review of the effects of computational thinking in pre-university education [63]. This article highlights the importance of computational thinking as an essential component in developing a reflective and critical education to solve everyday ICT problems. This paper presented the idea of ICTs, including gamification, as tools for computational thinking in STEM educational programs. However, this article indicates that such an approach has been studied and analysed in elementary, secondary, and post-secondary institutions, with no interest in university educational centres. This research highlights the lack of articles for less relevance of these methodologies in HEIs compared to other educational levels [71].

The third article is also a pre-and post-test experimental design, which was carried out for one group [64]. The methodology included a questionnaire and experimental interview based on undergraduates enrolled at an ICT course and focused on demonstrating the effectiveness of game-based learning considering the adaptability to the students’ learning [64]. This mixed approach aimed to determine the impact of gamification on university students’ performance and its effectiveness in their learning process. The results highlighted in this Turkish research showed a positive effect of gamification in the students’ motivation and engagement in their learning process. This article includes a highly relevant aspect of maintaining the students’ motivation and performance obtained via gamification. The researchers indicate that to achieve the previous goals via gamification, the role of the instructor should retransfer to the students. This shift positively affected the students’ engagement, but for a few, it negatively affected their performance.

The fourth article focused on a pre-and post-experiment design, based on 120 university students with no knowledge of SQL (Structured Query Language) [68]. The study included mainly Scottish undergraduate students from diverse HEIs (such as the University of the West of Scotland). The participants were divided into no-computing and computing students to determine the impact of gamifying to learn SQL as a coding language. The results of this Scottish experimental study pointed out that the students with the gamifying experience had better learning outcomes, such as performance and time to finish tasks.

The final research [65] was a systemic review that analyses the most researched gamification frameworks in the educational field. This systematic review identified 40 articles whose objective was to determine the impact of gamification in education. This research identified three prominent types of users: educators, designers, and researchers. This review indicated the importance of considering the main characteristics of the game elements and the focus and applicability in the higher education [65].

**Figure 3:**
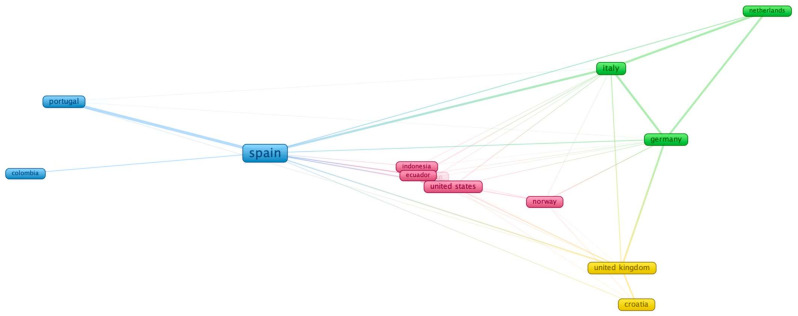
Collaboration among countries.

Additional to the results per country and top ten publications, the country co-authorship identified four clusters,190 documents with connections between ten countries and 26 links, indicating a low level of co-authorship. Despite this, the bibliographic coupling (Figure 3) identified more links (93 links) in 13 countries. The first cluster in pink was formed by five countries and 40 links (42.6%), which was led by the United States (US) with nine links and eleven documents. The countries from this cluster were present in 35 papers. The 2nd in green was formed by three countries, 23 links (24.5%), and 39 documents (18.5%), which was led by Italy with ten links and 21 papers. Three countries also formed the third cluster, which had 17 links and was presented in 113 documents, which was led by Spain (12 links and 98 papers). The last cluster was formed by two countries, with 14 links and 24 documents, led by the UK with ten links and 12 documents. Moreover, the highest organisation with more publications was the Polytechnic University in Valencia, with 59 documents representing 22.96%. According to the international ranking, these results could be explained by the fact that this University has a high impact in the engineering and interdisciplinary field [72].

These findings highlighted the new educational methodologies integrated into different European countries, with a high visualisation of Spanish researchers and HEIs. Most articles focused on reviewing or carrying out initial experiment designs, being the last published during the previous five years. Furthermore, the connective networking established how the countries with more publications were linked through the co-reference to other less representative countries, such as Spain with other European or South American countries [73,74,75]. Although the networking was small compared to other bibliometric studies [46], the networking showed how countries determined the influence of gamification and other ICTs in STEM education in HEIs [71,76]. Despite the links between countries, many studies indicated how gamification in HEIs was rarely compared to elementary or secondary educational institutions [63]; further research is needed on HEIs regarding the impact of gamification [73,74,75].

#### 4.1.3. Journals More Relevant in the Topic (RQ4)

**Figure 4:**
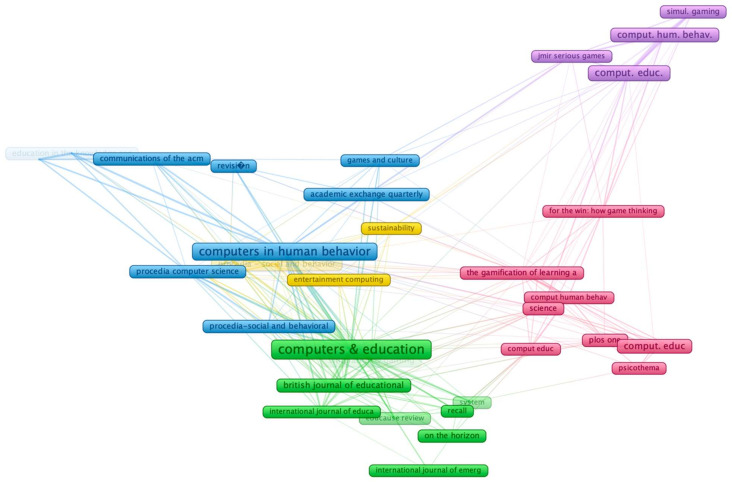
Co-citation between the journals.

The frequency of articles published in the ten top journals (Computers & Education, Digital Education Review, Cuadernos Canela, Frontiers in Psychology, and Revista Mediterranea Comunicacion—Journal of Communication with three documents each; and ARCHNET-IJAR International Journal of Architectural Research, ARTSEDUCA, Behavior & Information Technology, Computer Applications in Engineering Education, and Computers in Human Behavior with two documents each) showed 25 out of the total sample (9.7%), reflecting the low frequency of these journals. This low percentage was linked with the ratio of articles published in this area (*p* < 0.05), which was already established by previous studies [45,77]. Despite the low presence of these journals in the sample, these were connected to other journals through bibliographic coupling (Figure 4). Moreover, the cluster formed by the top ten journals had more links and citations, as happens with the second cluster led by *Computers & Education*, which had 32.8% of the citations (Table 4). The citations and links could be explained because these journals are indexed in the JCR and have JCI.

The results regarding the journal highlighted how much research on this topic is not published in journals, but the ones with higher relevance were available in critical journals with an association with the STEM area, mainly the engineering field. These findings indicated that the initial publications provided contextualisation and initial input into the new technology in the education [65]. The data are in sync with other technologies applied to the medical field, whose preliminary analysis or initial developments are presented [78].

#### 4.1.4. Determination of Sub-Topics Utilising the Keywords (RQ4 and RQ5)

**Figure 5:**
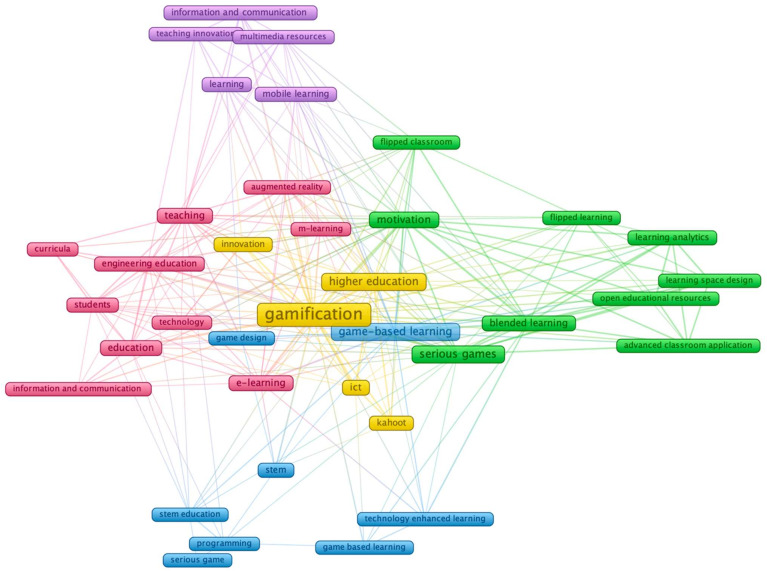
Co-occurrence of most common index terms per document. Note: the colours of the nodes indicate principal components of the data structure; the node size was scaled to the index keywords’ occurrences.

The co-occurrence of index keywords was analysed using a minimum of five nodes based on 736 keywords. Through this analysis, five clusters were identified, and 37 keywords were linked (Table 5). The concurrency indicated 237 links, with a strength of 633 and 505 concurrences (Figure 5). The first cluster, identified by pink, was formed by ten keywords with 55.7% of the links and 20.2% of the concurrences of 37 keywords determined. This cluster represented one of the main sub-topic topics based on technology as a new educational paradigm. The second in green represented 24.3% of the keywords and 22.2% of concurrences, which is the sub-topic motivation and new resources for teaching, such as flipped classrooms. The third cluster, represented by blue, reflected fewer concurrences than the previous clusters (14.8%), being the sub-topic focused on game design and STEM education. The fourth cluster, being in yellow, included the keywords with more concurrences (“gamification” with 108 and “higher education” with 31). The last cluster was formed by fewer keywords (13.5%) and concurrences (7.9%) was the topic based on mobile learning.

This analysis based on index keywords has shown how gamification has a beneficial impact on motivation and education in HEIs, which matches with the recent most cited article [69]. The clusters identified highlighted the theoretical frame of the ICTs in education at the beginning of the 21st century, whose impact continues to grow and deepen their effect [76,78]. However, the negative effect of these technologies, such as tiredness or loss of performance [79], seems to be missing from the current research, whose highlights focused on the positive impact on the students [80]. This topic seems to be in sync with the Goals for Sustainability and the US Health department [23,24,28], which highlighted how the ICTs are fundamental to social and healthy environments for the students. Therefore, these results showed how the teachers’ health or repercussion from using ICTs, anxiety or burnout syndrome, seems to be overlooked in contrast with the students’ [6,81]. Moreover, the inclusion of ICTs and gamification is more delimited to education, especially in the STEM field [20,71], highlighting the need for further research in other fields such as medicine or nursing [82].

### 4.2. Gamification in the Health Field (RQ6)

The studies focused on gamification in the health field have been analysed based on the results and the importance of gamification to improve motivation. There were 25 articles identified whose topic was gamification as a protective tool against healthcare issues, the mean of citations was 2.15 (SD = 2.05). The more frequent year of publication was 2020 (28%). The median was established in 2019, featuring 56% of conference proceedings, matching the previous results indicating how articles in this area are reduced. 

Moreover, the analysis of the co-citations about these documents highlighted that the more identified journals were Computers & Education (Q1 journal and had seven citations), Safety Science (Q1 journal and had six citations), Computers in Human Behavior (Q1 journal and had five citations), and Journal of Chemical Education (Q2 journal and had six citations) with percentiles over the 70th. These results also showed again that JCR played an essential role in the publications and citations of research articles, despite being so little indexed in this sub-topic (only two were indexed in JCR).

In addition, the co-occurrence of keywords identified only four common words in one cluster. The four words were “serious games”, “teaching”, “students”, and “engineering education”, which showed how this sub-topic is underdeveloped and continues to depend on engineering courses or frameworks before developing tools or current research. This interpretation matches the results from other articles produced in 2015 and 2016 that highlighted the use of gamification in healthcare but needed further research [82,83]. Moreover, data also highlighted how the year 2020, the year of the pandemic, resulted in a decrease in publications. This could be explained, since most of the recent articles were based on the contrast of the hypothesis, which is more challenging to carry out during lockdowns [84]. In this sense, a review [84] identified 11 articles that indicated the gamification strategies used, students’ assessment, and their motivation. The articles determined that motivation increased the learning process and allowed the students to be more perceptive to the lessons [84]. These results match an escape room’s initial evaluation as a gamification tool indicating their potential for teaching even postgraduate students [85]. The device was developed before the lockdowns and the experiment in Spain [85].

### 4.3. Implications and Limitations

This research methodology includes terms related to ICTs, gamification, and HEIs, excluding other keywords such as “schools”, avoiding the possible inclusion of documents based on the topic analysed. The keywords’ choice may limit the findings from the current research, since these are based on authors indexed and are not in the Medical Subject Headings. Therefore, the reduced number of articles could be explained by this selection and two databases selected (Scopus and WOS). Finally, the bibliometric analysis has a side effect, since it is based on less depth in the qualitative analysis and the overuse of quantitative metrics. Moreover, the Bibliometrix program could have been used to identify further cross-references. However, this research has intended to minimise this issue by combining various metrics, diverse metric programs, and understanding the field.

Despite the limitations, these findings have significant implications for understanding how the role of ICTs and gamification will evolve or continue in HEIs to create a sustainable and social educational environment. The results have indicated the relevant part of these technologies as motivational tools promoting healthy development from a more extended period than other studies focused on the last decade [45] or last five years [61]. Additionally, this analysis adds further information to the literature by elucidating the relevance of ICTs and specifically the gamification in HEIS and the future growth in experimental articles that could happen in the following decade [84], since most studies have presented an initial analysis [62] or review the previous studies carried out in early 2010 [65]. These results may help inform future investments in technology and education, which are more relevant now with the current pandemic, understanding the positive and negative effects of this methodology and the need for further intercontinental studies and collaborations between countries. In this sense, the bibliometric visualisations also provide an accessible means of communicating the key findings to researchers, policymakers, and students and teachers as members of HEIs.

## 5. Conclusions

This paper has argued that gamification seems to be a vital tool for creating adequate and sustainable HEIs, mainly through motivation and performance improvement. This applicability and relevance of gamification can enhance the learning process in any field, resulting in more relevance for scientific areas. However, the experimental studies seem to be carried out mainly in 2018 before the pandemic in took hold in European countries. Despite the positive effect, the results have indicated that little about the side effect or long-time impact has been analysed, since most studies were reviewed, and few empirical studies have been published. Moreover, based on the keywords and topics, most studies were based on the students, overlooking the teachers. Minor studies were based on teachers in HEIs, despite the top five most cited articles showing how previous articles to 2012 focused on educators or researchers. Moreover, previous studies indicated mental issues caused by excessive or incorrect use of ICTs and gamification, which teachers have not analysed. Based on the lack of experimental studies on the medium and long-term effect and impact of gamification in teachers, especially in STEM courses, the most significant development of gamification as an ICT tool for this field is yet to come.

In conclusion, this paper presented the global research patterns and current interests and identified the areas in which gamification, especially regarding the health sector, lacks depth. Additionally, the results have highlighted the need for more studies focused on the gamification effect on teachers or academics in the HEIs, whose overload can cause mental issues. Nevertheless, more work will need to be completed to determine the grade of inclusion or usage of gamification in HEIs as promoters of a healthy learning environment.

## Figures and Tables

**Table 1:** MeSH terms and description.

MeSH Terms	Description
Universities	Educational institutions providing facilities for teaching and research and authorised to grant academic degrees.
Learning	Relatively permanent change in behaviour that is the result of past experience or practice. The concept includes the acquisition of knowledge.
Education	Used for education, training programs, and courses in various fields and disciplines, and for training groups of persons.
Models, Educational	Theoretical models that propose methods of learning or teaching as a basis or adjunct to changes in attitude or behaviour. These educational interventions are usually applied in the fields of health and patient education but are not restricted to patient care.
Educational Technology	Systematic identification, development, organisation, or utilisation of educational resources and the management of these processes. It is occasionally used in a more limited sense to describe equipment-oriented techniques or audiovisual aids in educational settings.
Technology	The application of scientific knowledge to practical purposes in any field. It includes methods, techniques, and instrumentation.
Game Theory	Theoretical construct used in applied mathematics to analyse certain situations in which there is an interplay between parties with similar, opposed, or mixed interests. In a typical game, decision-making “players”, whom each have their own goals, try to gain an advantage over the other parties by anticipating each other’s decisions; the game is finally resolved due to the players’ decisions.
Games, Experimental	Games designed to provide information on hypotheses, policies, procedures, or strategies.

**Table 2:** Count of papers per country from the data.

	Country	Count of, Documents	Frequency		Country	Count of Documents	Frequency
1	Spain	93	36.20%	23	Belgium	2	0.80%
2	Italy	14	5.40%	24	Brazil	2	0.80%
3	UK	12	4.70%	25	Estonia	2	0.80%
4	Croatia	10	3.90%	26	India	2	0.80%
5	US	10	3.90%	27	Namibia	2	0.80%
6	Germany	9	3.50%	28	Philippines	2	0.80%
7	Norway	9	3.50%	29	Romania	2	0.80%
8	Indonesia	5	1.90%	30	Russia	2	0.80%
9	Japan	5	1.90%	31	South Africa	2	0.80%
10	Portugal	5	1.90%	32	Sri Lanka	2	0.80%
11	Hungary	4	1.60%	33	Sweden	2	0.80%
12	Ireland	4	1.60%	34	Taiwan	2	0.80%
13	Slovenia	4	1.60%	35	Turkey	2	0.80%
14	Bulgaria	3	1.20%	36	France	2	0.80%
15	Chile	3	1.20%	37	Argentina	1	0.40%
16	Colombia	3	1.20%	38	Canada	1	0.40%
17	Ecuador	3	1.20%	39	Costa Rica	1	0.40%
18	Finland	3	1.20%	40	Cuba	1	0.40%
19	Greece	3	1.20%	41	Czech Republic	1	0.40%
20	Peru	3	1.20%	42	Denmark	1	0.40%
21	Slovakia	3	1.20%	43	Others	13	5.20%
22	Australia	2	0.80%				

**Table 3:** The top ten most cited documents.

	Title	Year	Journal	Quartile and JCR Year of Publication	Thematic Area	Study	Country	Citations All Databases
1	Serious games and learning effectiveness: The case of It’s a Deal!	2012	Computers & Education	Q1 (2.775)	Computer Science, Interdisciplinary Applications	Article	Spain	146
2	Exploring the computational thinking effects in pre-university education	2018	Computers in Human Behavior	Q1 (4.306)	Psychology, Multidisciplinary	Article	Spain	76
3	Gamifying an ICT course: Influences on engagement and academic performance	2017	Computers in Human Behavior	Q1 (3.536)	Psychology, Multidisciplinary	Article	Spain; Portugal	76
4	An application of adaptive games-based learning based on learning style to teach SQL	2015	Computers & Education	Q1 (2.881)	Computer Science, Interdisciplinary	Article	Turkey	57
5	Gamification: a systematic review of design frameworks	2017	Journal of Computing in Higher Education	Q2 (1.517)	Education & Educational Research	Article	United Kingdom	56
6	Serious games and the development of an entrepreneurial mindset in higher education engineering students	2014	Entertainment Computing	-	Computer Science, Interdisciplinary Applications	Review	Spain	56
7	Training disaster communication by means of serious games in virtual environments	2011	Entertainment Computing	-	Medicine General and Internal	Article	Italy; Spain	45
8	Motivation, students’ needs and learning outcomes: a hybrid game-based app for enhanced language learning	2016	SpringerPlus	Q2 (0.982)	Multidisciplinary Sciences	Article	Germany	34
9	Learning style analysis in adaptive GBL application to teach SQL	2015	Computers & Education	Q1 (2.881)	Computer Science, Interdisciplinary	Article	Spain	28
10	Using Mobile Health Gamification to Facilitate Cognitive Behavioral Therapy Skills Practice in Child Anxiety Treatment: Open Clinical Trial	2018	JMIR SERIOUS GAMES	Q1 (3.351)	Medical Informatics	Article	United Kingdom	27

**Table 4:** Co-concurrency of journals that published on this topic.

Cluster	Links between Resources	Citations	Resources with More Links and Citations	Quartile and JCR in 2020	Category of JCR
1st	117 (50%)	116 (20.2%)	Science (17 links and 13 citations)	Q1 (47.728)	Multidisciplinary science
2nd	147 (62.8%)	188 (32.8%)	Computers & Education (29 links and 90 citations)	Q1 (8.538)	Computer science, interdisciplinary applications
3rd	94 (40.2%)	142 (24.7%)	Computers in Human Behavior (27 links and 57citations)	Q1 (6.829)	Psychology, multidisciplinary
4th	65 (20.8%)	53 (9.3%)	Journal of Computers in Education (16 links and 20 citations)	-	-
5th	34 (19.0%)	75 (13.1%)	Computers in Education (12 links and 37 citations)	-	-

**Table 5:** Main keywords used by the communities detected in the topic.

Cluster	Colour	Weight (%)	Connection between Clusters (Links per Keyword inside Each Cluster)	Keywords	Topic
1	Pink	27.0	132 (28.3%)	Students—teaching—engineering education—curricula—e-learning	Education through technological tools
2	Green	24.3	133 (28.5%)	Motivation—serious games—blended learning—flipped learning	Methodological educations impact on motivation
3	Blue	14.8	64 (13.7%)	Game based-learning—STEM-education	Gamification on STEM education
4	Yellow	13.00	86 (18.4%)	Gamification—Higher Education Institution—ICT	Gamification
5	Purple	8.97	52 (11.1%)	Mobile learning—multimedia resources	Education through mobile

## Data Availability

Data are available; please contact the authors.
